# The role of trust in engaging community-based task forces and agencies among minoritized communities during a public health emergency

**DOI:** 10.17269/s41997-025-01074-w

**Published:** 2025-06-30

**Authors:** Denessia Blake-Hepburn, Kadidiatou Kadio, Subrana Rahman, M. Hashim Khan, Samiya Abdi, Shaza A. Fadel, Sara Allin, Anushka Ataullahjan, Erica Di Ruggiero

**Affiliations:** 1 Dalla Lana School of Public Health, University of Toronto, Toronto, ON Canada; 2 Asthma & Airways Centre (TWH), University Health Network, Toronto, ON Canada; 3 Public Health Ontario, Toronto, ON Canada; 4 School of Health Studies, Faculty of Health Sciences, Western University, London, ON Canada

**Keywords:** Community engagement, Trust, Vaccine confidence, Community-based task forces/networks, Community agencies and/or community-based organizations, Public health emergencies, Engagement communautaire, Confiance, Confiance aux vaccins, Groupes de travail/réseaux communautaires, Organismes communautaires et/ou organisations communautaires, Urgences sanitaires

## Abstract

**Objectives:**

To investigate how task forces, networks, and community agencies engaged with faith-based, and ethnoracial communities to improve vaccine confidence and uptake of COVID-19 vaccines, and to understand the perceived enablers and barriers to the implementation of vaccine confidence and uptake in the Peel Region and Toronto, Ontario.

**Methods:**

Between June 2023 and March 2024, we conducted ten online focus groups with three task forces and six community agencies. We conducted four interviews with representatives from two task forces and one network. We used thematic analysis to explore respondents’ perceptions and experiences.

**Results:**

The data revealed that trust operated at interpersonal and organizational levels, which are mutually reinforcing. At the interpersonal level, members of the task forces, network, and ambassadors from community agencies drew on relationships with members of minoritized communities by addressing community concerns on their terms and using in-person, online, regular contact, and active listening approaches. At the organizational level, trust was facilitated through conducting outreach (i.e., vaccine promotion) at trusted and familiar locations (e.g., faith-based organizations). COVID-related information was better received from community representatives who were already known and trusted among community members. Common outreach strategies included door-to-door outreach; informational videos and sessions; mass awareness-raising campaigns; townhalls; and ethnic media and social media.

**Conclusion:**

Community leaders play an instrumental role in establishing and sustaining trust in vaccine promotion among community members. Trust established among community leaders and ambassadors enabled vaccine promotion efforts among minoritized communities. These findings may help to further strengthen community engagement for future public health emergency responses.

**Supplementary Information:**

The online version contains supplementary material available at 10.17269/s41997-025-01074-w.

## Résumé

**Objectifs:**

L’étude examine comment les groupes de travail, les réseaux et les organismes communautaires ont collaboré avec les communautés confessionnelles et ethnoraciales afin d'améliorer la couverture vaccinale et la confiance aux vaccins contre la COVID-19; identifie les facteurs favorisants et les obstacles au renforcement de la confiance aux vaccins dans la région de Peel et à Toronto, en Ontario.

**Méthodes:**

Entre juin 2023 et mars 2024, nous avons organisé dix discussions de groupes en ligne avec trois groupes de travail et six organismes communautaires, mené quatre entretiens avec des représentants de deux groupes de travail et d'un réseau. L’analyse thématique a permis d’explorer les perceptions et les expériences des répondants.

**Résultats:**

Les données ont révélé que la confiance opérait à la fois au niveau interpersonnel et organisationnel, ces deux niveaux se renforçant mutuellement. Au niveau interpersonnel, les membres des groupes de travail, du réseau et les ambassadeurs des organismes communautaires ont mis à profit leurs relations avec les membres des communautés minorisées pour répondre à leurs préoccupations dans leurs langues respectives. Ils ont également utilisé des approches interpersonnelles telles que le contact physique et en ligne, les contacts réguliers et une écoute active. Au niveau organisationnel, la confiance a été facilitée par la mise en place d'actions de sensibilisation (c'est-à-dire la promotion de la vaccination) dans des lieux familiers et dignes de confiance (par exemple, dans des organisations confessionnelles). Les informations relatives à la COVID provenant des représentants communautaires déjà connus et appréciés des membres de la communauté ont été mieux accueillies. Les stratégies de sensibilisation courantes comprenaient le porte-à-porte, des vidéos et des séances d'information, des campagnes de sensibilisation à grande échelle, des réunions et conférences publiques, ainsi que des émissions dans les médias ethniques et sur les réseaux sociaux.

**Conclusion:**

Les dirigeants et les responsables communautaires jouent un rôle essentiel dans l'établissement et le maintien de la confiance en ce qui concerne la promotion des vaccins au sein de leur communauté. La confiance établie entre les dirigeants communautaires et les ambassadeurs a permis de mener des efforts de promotion des vaccins au sein des communautés minorisées. Ces résultats pourraient contribuer à renforcer davantage l'engagement communautaire pour les futures interventions en contexte d'urgence sanitaire.

## Introduction

Community engagement is critical to bolster vaccine confidence, particularly among structurally entrenched groups during the COVID-19 pandemic (den Broeder et al., 2022). Vaccine hesitancy—driven by misinformation, historical mistreatment in healthcare, and distrust in government institutions —has hindered vaccination efforts, especially among faith-based communities and ethnoracial populations (Song et al., 2023). Building trust between individuals, communities, and public health institutions is crucial for engaging hesitant individuals and improving vaccine uptake (Ebrahimi et al., 2021).

Trust is pivotal to successful community engagement at interpersonal, institutional, and broader community levels, particularly in times of crisis (Davids et al., 2023). During the pandemic, ethnoracially minoritized populations experienced disproportionate impacts, including higher infection rates and systemic barriers to healthcare access, exacerbating historical inequities (Mude et al., 2021). The COVID-19 pandemic highlighted the critical role of trust in addressing these inequities (Chinekezi et al., 2023).

In this context, trust hinges on communities’ beliefs in the integrity of institutions and institutions’ deep understanding of the cultures, values, and needs of the populations they serve (Davids et al., 2023). However, past experiences of medical maltreatment and systemic racism have led many minoritized groups to distrust the health system — significantly hindering vaccine promotion efforts (Bogart et al., 2021). We intentionally use the term minoritized to emphasize minoritization as a socially constructed concept. A population group being minoritized does not imply a numerical minority but rather that they are actively socially excluded (Milner & Jumbe, 2020). However, we acknowledge that other terms are used in literature including vulnerable groups and ethnoracial minorities.

Collaborative relationships between organizations and individuals can strengthen emergency responses by leveraging earned trust and credibility (Ramsbottom et al., 2018). Community agents, such as faith leaders and grassroots organizations, play a crucial role in public outreach, serving as trusted intermediaries between public health institutions and communities (Corbin et al., 2021). These collaborations, often rooted in long-standing community structures like religious organizations and local non-profits, act as honest brokers, disseminate accurate information, and provide tailored health services, fostering deeper engagement and trust and highlighting the need for a multifaceted approach during the pandemic and in other situations (Ramsbottom et al., 2018).

Task forces and networks formed early in the pandemic in response to the disproportionate impact of COVID-19 on minoritized communities in Ontario. The task forces in Peel Region and Toronto consisted of multi-disciplinary members — including healthcare professionals, researchers, and community leaders who were commissioned or voluntarily convened for a specific mandate. Similarly, COVID-19 networks were informal, collaborative efforts focused on improving health outcomes for minoritized communities. While some initiatives were hyper-local, others extended their work across regions.

Despite the value of these community-based structures, many community and faith-based organizations (FBOs) often report feeling as underutilized resources by health system organizations, although they are recognized as trusted agents within their communities (Wilson et al., 2012). Public health partnerships established during emergencies also prove beneficial even outside of crisis situations, demonstrating the long-term value of these collaborations (Acosta et al., 2018; Stajura et al., 2012).

The Peel Region and Toronto were among the hardest-hit areas in Ontario, with high infection rates and low vaccine uptake due to access barriers and mistrust (Gill et al., 2022). Both have ethnoculturally and economically diverse populations, with more than half of their populations belonging to a minoritized group (Peel Region, 2021; Statistics Canada, 2024). In December 2020, Ontario launched the High Priority Communities Strategy (HPCS) allocating resources to lead community agencies to implement targeted interventions in the most affected neighbourhoods (Government of Ontario, 2020). Six agencies were assigned to Peel Region and five assigned to Toronto (Alliance for Healthier Communities, n.d.), each tailoring services to the needs of their communities (Government of Ontario, 2020).

While many studies focus on the importance of community engagement, there is limited research on how meso-level structures — such as community agencies, task forces, and networks — operated during the pandemic, particularly in settings such as Peel and Toronto with ethnoculturally and economically diverse populations. To the best of our knowledge, few studies have explored how these structures implement targeted vaccine promotion interventions in racialized and faith-based populations. Therefore, our study aimed to investigate how task forces, networks, and community agencies engaged with ethnoracial and faith-based communities to improve vaccine confidence and uptake of COVID-19 vaccines, particularly among vaccine-hesitant individuals from these communities; and to understand the perceived enablers and barriers to the implementation of vaccine confidence and uptake in the Peel and Toronto regions. Through this investigation, trust emerged as a critical enabler for fostering vaccine confidence among minoritized populations.

## Methods

We employed a qualitative research study design using focus groups and key informant interviews.

### Study area

The study was conducted in Ontario, Canada, mainly in the Peel and Toronto regions.

### Recruitment methods

We used purposive sampling to recruit participants from the task forces, network, and community agencies operating in the Peel Region and Toronto. Given this study was conducted in close collaboration with Peel Public Health, recruitment was only limited to the Peel Region for community agencies; its ethnoculturally diverse population was also disproportionately affected during the pandemic. Toronto is also mentioned because the catchment areas for the task forces and network included both Toronto and Peel.

#### Recruitment of task forces/networks

DBH identified and created a list of primary contacts for five task forces and one network which operated in the Peel Region during the pandemic and sought input from members of the research team (EDR, SAF, HK), familiar with these organizations. Invitations and consent forms were sent to the task forces and network of interest (see Table 1) by email in May 2023, with follow-ups continuing until August 2023. All groups agreed to participate.

**Table 1:** Data collection methods and participants

	Method of data collection	Number of participants
Community-based task forces and network operating in Peel Region and Toronto		
Black Scientists' Task Force on Vaccine Equity (BSTF; Toronto-based)	Focus group	*N* = 6
Canadian Muslim COVID-19 Task Force (CMCTF; Canada-wide)	Focus group 1	*N* = 4
	Focus group 2	*N* = 2
Kol Ha COVID Toronto Jewish Community COVID-19 Task Force (Toronto-based)	Interview	*N* = 1
Latin American COVID Task Force (LACTF)	Interview 1	*N* = 1
	Interview 2	*N* = 1
South Asian COVID-19 Task Force (SACTF; Canada-wide)	Focus group	*N* = 3
South Asian Health Network (SAHN)	Interview	*N* = 1
Lead community agencies (HPCS) in Peel Region		
Canadian Mental Health Association (CMHA)—Peel Dufferin Branch	Focus group	*N* = 2
Dixie Bloor Neighbourhood Drop-in Centre (DBNC)	Focus group	*N* = 5
Indus Community Services	Focus group	*N* = 4
Punjabi Community Health Services (PCHS)	Focus group	*N* = 4
Roots Community Services (RootsCS)	Focus group	*N* = 4
Wellfort Community Health Services	Focus group	*N* = 3
Total	*N* = 10 focus groups *N* = 4 key-informant interviews Total *N* of participants = 41	

Focus groups were initially planned to be conducted with eight to ten individuals from each task force and network to provide a comprehensive overview of how the task forces and network operated. Due to limited availability (e.g., many members returned to their full-time work), focus groups involved two to six participants, and individual interviews were conducted instead.

#### Recruitment of community lead agencies in the Peel Region

In August 2023, SAF and DBH contacted six community agencies (see Table 1) with the same recruitment materials. Some agencies did not immediately respond, and follow-ups continued into January 2024.

### Data collection

The team (DBH, KK, EDR, SAF, SA^1^, AA) developed a semi-structured question guide for review by our project knowledge user, Peel Public Health. The guide was then piloted among members of the research team and further modified based on feedback. We conducted ten focus groups (DBH conducted eight; MYS conducted two) and four interviews (DBH conducted two; MYS conducted two). In total, we had 41 participants: 22 from community agencies, 14 of which were community ambassadors (see Table 1).

Virtual focus groups and interviews were conducted using Zoom, between June 2023 and March 2024. Focus groups and interviews lasted approximately 50–70 min and followed a semi-structured ten-question focus group/interview guide (see Supplementary Material 1). Sessions were video and audio recorded with verbal consent from all participants before each focus group or interview. DBH transcribed eight, AK transcribed five, and SR transcribed one audio recording. Participants were sent a follow-up email to thank them for participation and to invite them to share documentation (e.g., a complete list of strategies implemented to promote vaccine confidence/uptake).

### Analytic approach

Data analysis was inductive, focusing on the meaning of the participants’ discourse rather than on a predetermined theory or conceptual framework (Braun & Clarke, 2006). We used thematic analysis (TA) to explore respondents’ perceptions and experiences: (1) familiarization with the data, (2) systematic coding of the data and development of a codebook, (3) development of an initial theme and consolidation of the codebook, (4) non-rigid coding guided by the codebook, (5) refinement of the codebook and development of a main theme, (6) report writing.

Data familiarization was carried out by two team members (DBH and KK) who examined noisy data from several reads, which were then transferred to NVivo 14 for coding (step 1). DBH and KK developed two preliminary codebooks based on coding four identical transcripts separately (step 2). DBH and KK identified the similarities and differences across the two codebooks and discussed and iterated the identified codes with EDR and then with other authors (SAF, SA^1^, AA) who provided subsequent feedback. DBH then drafted a consolidated codebook which went through a thorough review process with KK to ensure each code was defined (step 3). DBH and KK proceeded to code the remaining ten transcripts focusing on the content of the respondents’ speech. The coding identified initial themes and sub-themes related to the processes of community engagement of the task forces, network and community agencies, as well as themes related to trust (step 4). An in-depth reading of the content of codes enabled DBH and KK to refine the codebook (moving some content, merging others), develop the main themes and sub-themes and reformulate them (step 5). This article reports on findings related to trust at multiple levels: interpersonal and organizational (step 6). We define interpersonal trust as one person’s belief in the reliability, honesty, and abilities of another person in a relationship (Essex et al., 2022). “Organizational trust relates to people investing trust in a system or institution (as distinct from a person), such as a hospital, a medical clinic, or a cancer screening program. Trust in organizations has been strengthened by recognition of their community roots” (Ward, 2017).

## Results

Trust and its association with vaccine confidence was a recurring theme across all data. The data revealed that trust operated at interpersonal (trust in the individuals serving on the task forces, network, community agencies) and organizational (trust in the task forces, networks and community agencies as credible structures) levels, which are mutually reinforcing. Community representation was also linked to trust such that having different cultures and languages represented on the task forces, networks, and community agencies made people from minoritized communities (e.g., Black, South Asian), belief-based groups (e.g., Jewish, Muslim), marginalized groups (e.g., farm workers, migrants), and other populations with intersectional social identities (e.g., Muslim women) feel more comfortable interacting with these community structures. Interpersonal interactions enabled community ambassadors to interact with their communities, learn and adapt their strategies to their communities’ needs and fears, and combat mis- and disinformation about vaccines, thereby building trust both in the ambassador and in the organizations they work for. Across the task forces, networks, and community agencies, outreach strategies based on a bottom-up and non-judgmental approach were applied to establish rapport and trust with multiple population groups (e.g., minoritized, faith, marginalized).

### Trust and vaccine confidence

#### Trust at the interpersonal level

Trust was predominantly expressed at the interpersonal level, in which community ambassadors, faith leaders, task force, and network members reported promoting and brokering trust with vaccine-hesitant individuals from minoritized, marginalized, and faith-based communities. The results showed that trust is mainly relational and dynamic. Interpersonal interactions over time with people representing an organization with which community members can identify culturally (community representation), helped to reinforce trust. This trust, first interpersonal and then organizational, fostered acceptance of COVID-19 vaccine messaging.

Community ambassadors were community members who represent the culture and languages of their communities and were thus perceived as trusted sources of information. The community agencies had teams of community health ambassadors who were paid, and part of a broader team of influential liaisons. Community ambassadors engaged in their own relationship-building processes; addressed community concerns with updated COVID-19 information; used in-person regular and accessible contact; engaged in active and patient listening; and responded to community needs on their own terms.

The task forces and network were formed prior to the HPCS and the funding of community ambassadors. For the task forces and network, members were building and establishing trust for the first time, with people that were not already connected to the task forces and network; this differed from community members who may have already been connected to a particular community agency and had some buy-in in that regard. The task forces and network worked alongside community agencies to address vaccine mis/distrust as a response to a gap in the provincial and local public health response.

As trusted members in their communities, faith-based leaders were a liaison between the task forces, network, community agencies, and their communities. The task forces and network reached out to leaders to identify their needs and possible ways to support. Faith leaders had to navigate changes in religious meetings and holidays— and these leaders who were trusted and respected in their community were key to helping their communities understand temporary restrictions from a religious perspective. Beyond restrictions, many were helpful in becoming ambassadors themselves and promoting vaccine uptake as a faith-compatible individual and collective responsibility.

Interpersonal interactions enabled the ambassadors, task forces, and network members to interact with their communities, to learn and adapt their strategies based on their communities’ needs and concerns, thereby building trust both in the ambassador and in the organizations they work for. Interpersonal interactions between community members and staff (e.g., community ambassadors, faith leaders) from community agencies that people already trust and feel connected to strengthened confidence in their vaccine promotion work.

#### Trust at the organizational level

Organizational trust was enabled because community agencies were already known and frequented by community members to satisfy and fulfill a variety of needs (e.g., food access, employment, and financial needs). Organizational trust was also manifested through familiarity with existing community organizing work, which helped build trust in the organization and enhanced receptivity to their COVID-19 vaccine messaging.

Community’s familiarity with community agency’s services and community work helped to facilitate trust. Thus, when staff would go to different outreach locations to promote vaccines, there was a sense of familiarity with their work which allowed for ease of activities. Ambassadors found that people were more open to listen because they were familiar with the agencies:

I will say that a lot of the local businesses and community members that we’ve built trust with already, it’s really easy to sometimes promote the activities we do now because they see us and they say, ‘oh, look, it’s people from X [organization]’. And they’re open to whether it is hanging up flyers in their shops to promote our organization, or upcoming activities, or for them just to come out and participate […] They’re open to listen because they know about us. They know that we mean good intentions and they trust us. (Focus group 8)

Many task force and network members already had community buy-in because of their experience with community organizing and being connected with senior leadership who are very respected:

We have a lot of experience, not we as in individuals, but community organizing and running our own community institutions, and the other big thing is that there's a whole literature on medical X law with respect to medical ethics and vaccines […] Large organizations as well as individuals would write these things and we had access to them, and people had great respect for them. When someone wrote something like that it was quite important and influential, and it wasn't our first kick at this can. (Interview 3)

#### Community representation and trust

Community representation was a key driver in building interpersonal and organizational trust because community members saw their culture and language reflected in the membership of the task forces and network, including health professionals, scientists, community and religious leaders, and ambassadors, and in the efforts made by community agencies. For instance, a community agency (e.g., CMHA) primarily serving South Asian populations also ensured they had ambassadors that spoke several South Asian languages (e.g., Hindi, Urdu, Punjabi) which resulted in the information being better received. These individuals engaged in proximity interactions with minoritized communities, including specific racialized cultural groups (e.g., Black, African, Caribbean, West and South Asian), belief-based groups (e.g., Christian, Muslim, Jewish communities), marginalized groups (e.g., farm workers, migrants), and other populations with intersectional social identities (e.g., Muslim women). These populations were prioritized in vaccination promotion strategies because they were identified as populations being disproportionately impacted by COVID-19 or being missed in public health responses.

Community representation built credibility within the task forces, networks, and community agencies as trusted sources of information. Task force and network members held important positions of knowledge, power, and expertise (e.g., senior public health physicians) while also being members of the communities they were reaching:

Even if there were myths or hard hitting questions, we came at them with that expertise and that expertise is one of the ways that we gained trust as well. So, representation was obviously a big thing. Having a group that represented Black people was important for this group especially. But it was also the caliber of expertise that we had on this group. So, it was Black faces, yes. But, very wise, experienced Black faces at that. (Focus group 1)

The visibility of task forces and network members as familiar faces present in familiar spaces contributed to trust. They were present on ethnic media (i.e., media produced by and for ethnic communities), and in clinics, which contributed to the success of clinic attendance:

What was important for the success of the task force is also making sure that the members of the task force, who were physicians, who were trusted in the community, who were seen on these ethnic media channels were also in the clinics…providing vaccines. (Focus group 2)

In short, community members were more receptive to COVID-19 vaccine information coming from the task force, network members, and community agency staff who shared their cultural background and spoke their languages. Community representatives in the task forces and network also held positions of knowledge, power, and expertise, which helped to establish the credibility of these organizations.

### Approaches to build trust

#### Proximity interactions

Community ambassadors played a critical interface role in part because individuals were more willing to listen to a familiar and trusted member of their community than an authority figure.

Trust building first began with ambassadors arming themselves with COVID-based knowledge to combat misinformation and mis/distrust in COVID-19 vaccines. Ambassadors hired by community agencies frequently attended educational sessions on COVID-related information and vaccines, for example through Health Commons Solution Lab, a community organization dedicated to co-designing solutions with the community. They took the initiative to adapt their organizations’ strategies to the realities on the ground. Some of the task forces and networks also provided their own in-house expertise.

Ambassadors maintained a constant presence in the community at various outreach locations. In-person contact was particularly useful for building trusting relationships to promote the vaccine and support community members to access wrap-around services (e.g., grocery, financial) so that they could isolate safely at a designated location, prior to the availability of vaccines. This allowed vaccine-hesitant individuals from minoritized, marginalized, and faith-based communities to feel safe and comfortable to openly address their concerns about vaccination — for example:

The fact that we were consistent, we would be like, just because they said no the first time that didn’t mean that we would stop going within the community. We might not go to the same house, but they will see us walking around in the neighborhood, going to the other places and talking to them. And…over the time we built that trust and rapport because we were consistent in our approach…wherever we were able to help the community, we stayed on [sic] those places, got hold of, understood how we can support more. (Focus group 6)

Their constant presence helped to create a sense of familiarity among their community members who became more comfortable reaching out to ambassadors. Ambassadors were a bridge between the public health system and the community. They built deeper personal relationships with community members in a way that the health system was not able to, which also contributed to trust-building among community members.

I think getting in bits and pieces, when we get to know that people are getting back to us, they are calling us and they’re reaching out to us to know more about it. I think that was one of the best thing to measure that, yes, it was really helpful that we are out there in the community and we have become a kind of bridge between the health system and the community that people are able to reach out to health system through us. (Focus group 9)

Ambassadors, faith leaders, task force, and network members utilized various conversational approaches which helped facilitate trust and rapport among community members. For instance, respondents preferred to have one-on-one conversations with community members to address their specific concerns and needs. These respondents reported the importance of patience and active listening:

We did a lot of active listening when we were trying to get people to be vaccinated in ways that some of them didn’t want to leave their homes. Some of them came to the parking lot in their cars and we were able to get the nurses to go out and offer the vaccination. We did a lot of active listening which translated into actions that today many people still remember those acts of goodness, because they see it as they weren’t abandoned, and they had a voice. (Focus group 3)

Staff from community agencies were asked to address other needs beyond vaccination. They would redirect these individuals to internal services and supports (e.g., employment support, settlement linkages, financial, grocery and food support) to address these needs. Some often came back to the ambassadors (after their needs had been met on their terms) to find out more about the vaccine, and often to be vaccinated:

‘Oh, I’m not ready for the vaccine. You guys are promoting vaccine, but I need job. I got laid off’. It’s like, ‘okay, let’s start there first’ because at X [organization], we’re a multi service agency, we provide employment and all that settlement support. We met a lot of people who do not speak English and been here for years. We connected them with settlement link… And once they had this, once we have this relationship with the client, they come and tell us, ‘Okay, now I'm ready. Let’s take this’ (Focus group 7)

By ensuring well-being was the priority, ambassadors reinforced trust and reduced mis/distrust and hesitation among community members. The task forces and networks also utilized a similar approach; they would make provisions (e.g., transportation and food at vaccine clinics) for hesitant individuals from minoritized, marginalized, and faith-based communities and people experiencing access issues to clinics. They also used their own in-house expertise to host accessible vaccine clinics (e.g., clinics at FBOs), employ other healthcare professionals from partnering hospitals to help administer vaccines, and respond to other community needs.

#### Awareness-raising based on community needs and expectations

Across organizational and interpersonal levels, the task forces, networks, and community agencies used a bottom-up and non-judgemental approach to facilitate trust, including outreach strategies such as town halls and the use of ethnic media.

Respondents reported outreach strategies, including door-to-door outreach; informational videos and sessions to address questions and concerns; mass awareness-raising campaigns (e.g., ThisIsOurShot); statements from community leaders endorsing the vaccines; and social media to share information. Vaccine-hesitant individuals already did not trust information coming from the health system and government because they did not feel seen as a priority. Respondents shared that some individuals had discomfort or negative experiences interacting with the healthcare system (i.e., seeing physicians) prior to the pandemic and these issues continued to be evident during the pandemic, including not being able to speak English, not being covered by OHIP, and not wanting to interact with police who may be present (e.g., hospital buildings). Reportedly, trust in authority bodies was a significant issue.

The task forces, networks, and community agencies acknowledged the historical exploitation minoritized communities have faced, including mistreatment by the health sector, and respected the choices of individuals who refused the COVID-19 vaccine because of mis/distrust. For instance, one task force applied a non-judgmental approach to their events such that people who were vaccinated and unvaccinated were welcome to attend events (e.g., health fairs). Virtual and in-person town halls provided a safe space for people to speak about historical exploitation and their vaccine hesitancy and to address their concerns.

The task forces and network also leveraged ethnic media (e.g., radio, print media) to deliver townhalls, education, and information in the languages that are already used in ethnic media and combat misinformation, especially within South Asian and Latin communities. Respondents reported these were already trusted sources of information — therefore, they were better able to understand and receive information coming from these sources.

## Discussion

Existing literature, including systematic reviews, has examined the broader role of trust during public health crises and how both the nature of emergencies and the actions of organizations working in these contexts influence people’s trust (Sopory et al., 2022; Zhai et al., 2022). While there is growing literature on the community engagement mechanisms used by health systems to change the vaccine-seeking behaviours of communities in the context of public health emergencies, our study centres on the perspectives of communities, including the role of trust as an essential contributing factor. This study fills a gap in the trust-building processes of community engagement at a meso-level (community-based task forces and organizations) and explores how community-based task forces and networks and community agencies facilitated trust among their community members in the Peel Region and Toronto during the COVID-19 pandemic.

This study elucidated different approaches to building trust and levels at which trust operated among and between community-based task forces and community agencies to promote vaccine confidence. Interpersonal trust was shown to be a precursor to organizational trust. At the interpersonal level, respondents revealed the importance of establishing their own relationships and connections with community members through addressing community concerns and needs on their own terms, through in-person and regular contact, and active listening. At the organizational level, respondents shared that they garnered trust by accessing hesitant individuals from minoritized, marginalized, and faith-based communities through trusted and familiar locations (e.g., FBOs). COVID-related information was better received from community representatives who were already known and trusted. Respondents reported common outreach strategies (e.g., door-to-door outreach; mass awareness raising campaigns; ethnic media and social media) informed by a bottom-up and non-judgmental approach.

Our study supports existing social theories on trust which argue that trust is “rooted in a combination of interpersonal behaviours and institutions that underpin those behaviours” (Gilson, 2005). Interpersonal trust is a precursor for organizational trust; for instance, individuals may need to trust the physician, who is the access point, before they trust the medical system. However, public trust has been on the decline in Western health care systems and has been linked to decreasing confidence in science and experts (Ward, 2017). Therefore, trust requires a process of negotiation to be worked on and won (Giddens, 1991). The study sheds some light on the mechanisms of cooperation, collaboration, and governance that allowed for this highly effective engagement approach to strengthen trust among actors working within and with the system. Although beyond the scope of this paper, it remains to be seen how long these collaborative mechanisms will continue to persist in the current context.

The process of building trust is dynamic and operates on a continuum, in that trust can change over time and is not a linear binary variable (Gilson, 2005). An individual’s level of trust may be informed by available evidence like past experiences with the medical system or community agencies that provide wrap-around supports and services (Luhmann, 1979). Our data showed that pre-existing relationships and experiences helped to inform people’s responses towards vaccine promotion work during the pandemic, emphasizing the importance of interpersonal trust building as a strategic priority for future public health emergencies.

Our findings align with other study results that are part of our overall research project. We conducted interviews with Ontario public health units (PHUs) to investigate their work with FBOs to improve vaccine confidence among minoritized communities during the COVID-19 pandemic (Kadio et al., 2024). Task force, network members, and staff from community agencies reported similar facilitators as PHU respondents to their vaccine promotion work, including active listening, pre-existing relationships between FBOs and PHUs, respecting different beliefs and opinions on the vaccine, having representatives on these groups who are also members of the communities, and conducting outreach in familiar and trusted places (Kadio et al., 2024). These findings reinforced the importance of establishing trust with community members at interpersonal and organizational levels and the inclusion of community representatives in outreach work.

At the organizational level, decision-makers, including public health and the government, should aim to take a community-centered approach which prioritizes listening to community voices from minoritized communities (Quinn & Andrasik, 2021; Ramsbottom et al., 2018). Institutions should aim to address inequalities and inequities related to health, wealth/income, and education that were exacerbated during the pandemic. Tackling inequalities and inequities must occur on an ongoing basis not just within the context of public health emergencies. This is particularly important given that many people mis/distrusted government messaging and the vaccines during the pandemic (Baack et al., 2021) due to inconsistencies with prioritizing vulnerable communities prior to the public health emergency, as reported by respondents. Therefore, institutions must seek to acknowledge and address past and current historical injustices.

### Limitations

Our study is not without limitations. We experienced challenges with recruitment and participation for a variety of reasons including most of the task forces and the network disbanding and healthcare professionals returning to their full-time jobs, difficulty getting people’s schedules aligned or reaching the appropriate contacts. Therefore, some focus groups were not as representative of their organizations or task force and network. While this theme did not emerge in our data, we recognize that trust-building processes can be influenced by gendered dynamics and should be the subject of future studies (Collischon & Patzina, 2022). Another limitation (due to feasibility) is that our data only captured the perspective of trust from community leaders (i.e., task force and network members, staff from community agencies) which may not comprehensively reflect the opinions of individuals from their respective communities. However, our study still contributes a partial perspective as members of these task forces and network, and community agency staff were representative of the communities they serve.

## Conclusion

The lack of trust in the health system and the COVID-19 vaccines emerged early in the pandemic. The role of trust surfaced as a key finding contributing to successful community engagement during the COVID-19 pandemic and playing out at interpersonal and organizational levels. Our findings further suggested that task forces, networks, and community agencies can act as a meaningful bridge between community members who have been historically marginalized and public health agencies.

## Contributions to knowledge

What does this study add to existing knowledge?

- This study fills a gap in knowledge of what is known about the trust-building processes to support community engagement, drawing on the experiences and perspectives of actors working at a meso-level (community-based task forces and agencies) mainly in the Peel Region and Toronto during the COVID-19 pandemic. Peel Region and Toronto were among the hardest hit regions during the pandemic.

What are the key implications for public health interventions, practice, or policy?

- Trust-building is a dynamic ongoing process that should first start at the interpersonal level.
- Public health and other health system actors should aim to involve community representatives and leverage existing community networks and relationships as an ongoing practice in public health work.

## Supplementary Information

Below is the link to the electronic supplementary material.

Supplementary file1 (DOCX 37 KB)

## Data Availability

Full transcripts cannot be shared publicly due to potentially identifying information. The content and words of respondents in focus groups and interviews could potentially be used to identify individuals.

## Code availability

Codebook is available on request from the authors.

## Declarations

### Disclaimer

The funder had no role in the design, collection, analysis, and interpretation of data, nor in the writing and decision to submit this manuscript for publication.
