# Supplementary material for: The role of trust in engaging community-based task forces and agencies among minoritized communities during a public health emergency
Source: Can J Public Health. 2025 Jun 30;117(2):214–24. doi: 10.17269/s41997-025-01074-w (PMC13076815; doi:10.17269/s41997-025-01074-w)
Supplement: Supplementary file 1 — Supplementary file1 (DOCX 37 KB) [file 41997_2025_1074_MOESM1_ESM.docx]

**Focus Group/Interview Guide for Task Forces/Networks/Community Agencies working with Faith, Racial and Ethnic Communities**

**Sample questions from focus group/interview guide:**

1. *Please tell us about your role in the Task Force/Network/Community Agency and how long have you been involved.*
2. When was your Task Force/Network/Community Agency established? (Probe for its origins, reasons, motivations, initiators)
3. *Now we would like to discuss your Task Force/Network/Community Agency’s activities*:
4. Please take a moment to recall a collaboration between your Task Force/Network/Community Agency and other organizations (e.g. public health unit, other faith-based groups) to promote vaccine uptake (e.g., vaccine drives, education sessions, Q&As, et cetera). What meaningful activities did the Task Force/Network/Community Agency perform?
5. Was your Task Force/Network/Community Agency able to promote trust in vaccines?
6. How did you measure the success/impact of your activities? Can you give examples?
7. *We have reached the end of our focus group/interview:*
8. Would you be able to share any relevant documentation with us after this focus group/interview?
